# Capital structure, business model innovation, and firm performance: Evidence from Chinese listed corporate based on system GMM model

**DOI:** 10.1371/journal.pone.0306054

**Published:** 2024-06-21

**Authors:** Xu Tian, Yan Wang, Umar H. A. Kohar

**Affiliations:** 1 Science and Technology Finance Key Laboratory of Hebei Province, Hebei Finance University, Baoding, Hebei, China; 2 Faculty of Management, Universiti Teknologi Malaysia, Johor Baru, Malaysia; 3 Faculty of Management, Hebei Finance University, Baoding, Hebei, China; 4 Faculty of Business and Communications, INTI International University, Putra, Nilai, Malaysia; Ho Chi Minh City Open University, VIET NAM

## Abstract

This paper aims to verify the impact of capital structure on business model innovation and firm performance and the mediating effect of business model innovation. We use the data of the Chinese growth enterprises market (GME) listed high-tech firms from 2016 to 2022 as a dynamic panel data model with the system–generalized method of moments (sys-GMM), adopting return on asset and earning per share as firm performance. Our results show that capital structure has a lag effect on firm performance. The total debt ratio in the last period has a significant non-linear impact on the performance and business model innovation level of nowadays, presenting a U-shaped relationship. The first-order lag short-term debt ratio positively improves firm performance. Business model innovation significantly promotes better firm performance, and business model innovation does exist in the mediating effect between enterprise capital structure and its performance. These results remain robust to different sample sizes or proxy variables. This paper proposes some suggestions for firm operations and government policies based on the findings.

## 1. Introduction

In the last decades, the Chinese economy has expanded ten times, and the achievement is remarkable to the world. As the National Bureau of Statistics shows, in 2022, the gross domestic product (GDP) exceeded 18 trillion US. Dollars, for the first time, are firmly ranking second in the world. Some firms perform better because of the business model innovation (BMI) and capital investment support [1, 2].

The relationship between capital structure (CS) and firm performance (FP) has been a hot research topic in the academic field. Capital structure is an essential factor that a firm can adjust and control by itself, affecting performance [3–8]. Scholars have used many methods such as multiple regression, structural equation modeling, and data envelopment analysis, taking long-term and short-term leverage ratios and large shareholder ratios as indicators, but have obtained completely different research conclusions. These research results include positive relationships [9–12], negative relationships [4, 13–16], and complex relationships [3, 17–20]. In general, the relationship between capital structure and firm performance is complex and is affected by various factors.

With the advancement of technology and the arrival of the mobile internet era, business model innovation is also profoundly impacting the firm performance. It can be seen countless institutional investors take advantage of the new rules of the competition and the power of the capital market to transform the business models of target enterprises, creating a variety of different business models, such as sharing models, community models, free models, long tail models, and platform models. This has changed the performance level of firms and realized investment appreciation by improving the value of firms in the capital market. Firms have also achieved long-term development.

Since 2001, when Amit and Zott began studying e-commerce platform models [21], scholars have researched business models and business model innovation [22–31]. Overall, the research on the relationship between business model innovation and firm performance is still in the early stages. However, most existing research suggests that business model innovation is a promising approach that can help firms to improve their performance.

In recent years, scholars have increasingly paid attention to the role of business model innovation as a mediating mechanism to explain the relationship between firm performance and a variety of factors, including technology [32–34], university spinouts [35], value chain activities [36], novel products and services [37], organizational adaptation and resistance [38], and integrative capability [39].

Since capital structure is essentially a matter of firm control, and business model innovation requires sufficient influence to make continuous adjustments and changes at the organizational or strategic level, it is natural to study the transmission mechanism of business model innovation in the relationship between capital structure and firm performance. Therefore, this paper aims to use data from the Chinese growth enterprises market (GEM) listed firms from 2016 to 2022 to explore how capital structure affects business model innovation and how this, in turn, affects firm performance.

The paper proceeds as follows. The literature review section reviews the researches on capital structure, business model innovation, and firm performance. Methodology and Data Source section explains the research methodology, sample and data source. Results section reports the regression results. Discussion section discusses the every results. Conclusion section provides the conclusion, implications, limitations, and future works.

## 2. Literature review

### 2.1 Capital structure and firm performance

Capital structure can be divided into two perspectives: narrow and broad. In the narrow sense, capital structure refers to the composition of debt and equity financing of a firm, focusing on the firm’s financing structure with the total debt divided by total assets [7, 13, 40–42]. Leverage is the most widely used indicator of capital structure [41, 43–46]. In a broad sense, capital structure includes the composition of long-term and short-term debt ratios and the firm’s top shareholders’ equity ratio [20, 47, 48]. Over the years, scholars have worked hard to find the optimal capital structure for firms and identify the relevant influencing factors in choosing different capital structures. They hope to provide firms with a unified conclusion that can guide them to adjust their capital structure and thus enhance enterprise value. This has led to a wealth of research results.

Regarding the impact of capital structure on firm performance, scholars have proposed the following theories, such as the Modigliani–Miller theory [49, 50], the agency theory [51], the pecking-order theory [52], the trade-off theory [20, 53], the market timing theory, the signaling theory, the efficient-risk hypothesis and the franchise value hypothesis [6, 54, 55]. These theories and hypotheses provide theoretical support for subsequent complex capital structure research. Scholars also constantly use the corresponding theories to explain their research findings and eventually form different research conclusions.

Some scholars conclude they are positive relationships [9, 12, 56–61]. For example, [62] used the generalized method of moments (GMM) and applied data from 367 firms in growth markets to construct a model by indicators such as debt ratio and return on investment, obtaining a positive conclusion. [63] used the ordinary least squares with data from 493 non-financial firms in different industries to investigate the relationship between total debt rate and firm ROE/ROA, they found a positive conclusion. Moreover, some scholars find they are negative relationships [64–69]. For the conclusion of a negative relationship, a broader range of indicators and scope is used [15, 70]. [42] used WarpPLS analysis to study the relationship between debt-asset ratio, debt-equity ratio with ROA, ROE, and Tobin’s Q for 182 publicly listed manufacturing firms and obtained negative findings. Meanwhile, some other scholars believe that capital structure and firm performance have a more complex relationship [71–76]. It is not linear but possibly U-shaped, inverted U-shaped, or different for different indicators [71, 77–80], even with no links [81–84]. Furthermore, Table 1 summarizes the research findings on the relationship between capital structure and firm performance.

**Table 1:** Capital structure and firm performance.

Capital Structure and Firm Performance	Literature
Positive	[12, 56–59, 61, 85]
Negative	[4, 64–70]
U-shaped	[75, 76]
inverted U-shaped	[17, 71–74, 78]
No links	[43, 81–84]
Different for different indicators	[8, 18, 80, 86]

### 2.2 Business model innovation and firm performance

Scholars have gradually paid attention to the concept of business model since 2001 and have distinguished the concept of business model from existing concepts such as strategy and profit model, considering business model as the overall operation of a firm, which relies on operations to go beyond competitors and provide value to customers [21, 87, 88]. Since 2010, scholars have gradually shifted their research focus from business models to business model innovation. This is because innovation usually leads to better performance. Therefore, scholars are more interested in the process and manner of business model innovation [33, 89, 90]. In practice, firms hope to improve business performance through innovation in the business model. Whether it is efficiency-based or novel-based business model innovation, it often involves the adjustment and change of elements to achieve business model innovation, thus achieving improved performance [24, 91, 92]. Research on business model innovation has evolved from a linear to a complex analysis, from an internal to a holistic perspective, and from an independent to a multidimensional approach.

The research on the relationship between business model innovation and firm performance has become more complex and specific as the elements considered and the methods used in the study have become more sophisticated. Scholars have conducted case studies, hierarchical regression, partial least squares, structural equation modeling, and fuzzy sets qualitative comparative analysis to study firms in manufacturing, technology, insurance, and fashion and apparel industries in China, Sweden, Italy, and Southeast Europe [32, 34, 93–97]. Most findings show that business model innovation has a significant positive relationship with firm performance.

[39] used structural equation modeling to analyze data from 165 Chinese firms and found that the relationship between business model innovation and firm performance is not simply linear but influenced by many factors. [98] used two-step cluster analysis to analyze data from 72 international construction contracts and found similar results. [95] conducted a case study of a mobile technology provider in the technology industry. They found that the relationship between business model innovation and firm performance was complex, with the impact of business model innovation depending on many factors, such as the industry, the firm’s competitive position, and the strategic fit of the business model innovation. [94] conducted a case study of an insurance firm and found similar results. Table 2 summarizes the research findings on the relationship between business model innovation and firm performance.

**Table 2:** Business model innovation and firm performance.

BMI and Firm Performance	Literature
Positive	[22–24, 32, 34, 92, 93, 96]
Complex or U-Shaped	[25, 39, 94, 95, 98]

### 2.3 Mediating role of business model innovation

Business model innovation has a relatively large scope and degree of impact. Scholars have used case studies [32, 36, 37], regression analysis [39], structural equation modeling [34, 38], and literature review methods [33] to analyze the mediation effect of business model innovation. The research subjects include Xerox firm [32], 150 peer-reviewed scholarly articles [33], 165 Chinese firms [39], an agricultural information service provider in India [37], and 104 organizations from different industries [38].

Scholars have studied the role of business model innovation as a mediator between technology [32, 33], value chain activities [36], and other different variables with firm performance. It was found that business model innovation mediates in improving firm performance. Table 3 summarizes the research findings on the mediation effect of business model innovation on firm performance.

**Table 3:** Mediation effect of business model innovation on firm performance.

IV	DV	Research method	Literature
Technology	Economic value	Case study	[32]
University Spinouts	Firm performance	Case study	[35]
Value chain activities	Firm performance	Case study	[36]
Technology	Economic value	Literature reviews	[33]
Novel products and services	Firm performance	Case study	[37]
Organizational adaptation/resistance	organizational performance	SEM	[38]
Integrative Capability	Firm Performance	Regression	[39]
Technological innovation	Success performances	SEM	[34]

Our study completes the literature by surveying the impacts of capital structure on firm performance using a dynamic panel data model and sys-GMM. For further analysis, we examined the impact of capital structure on business model innovation and the influence of business model innovation on firm performance in China. The literature supports a common finding on the role of capital structure on firm performance and business model innovation.

## 3. Methodology and data source

### 3.1 Methodology

Firm performance is not only affected by external factors but may also be closely related to the firm’s past performance level and present a certain stickiness. The specific model settings are as follows:

$$y_{it}=\alpha_{1}y_{i(t-1)}+\alpha_{2}y_{i(t-2)}+\beta^{\prime }(L)x_{it}+C_{it}+\lambda_{t}+\eta_{i}+\varepsilon_{it}$$ (1)

Where *t* = 1, …, T, and *i* = 1, …, N. T and N denote the time and the firm, respectively. *y*_*it*_ is the dependent variable, *y*_*it*−1_ and *y*_*it*−2_ are the lagged of the dependent variable. (*L*)*x*_*it*_ represents all independent variables and their lagged terms, and 2 *C*_*it*_ are control variables. *λ*_*t*_ is the unobserved time-invariant effect, *η*_*i*_ is the unobserved firm-specific effect and *ε*_*it*_ is the error term.

In different models, *y*_*it*_ represent the level of firm performance (FP) and business model innovation (BMI). The firm performance is captured by the return on asset (ROA) [70, 72, 99–101] and earnings per share (EPS) [20, 44, 54, 77, 85]. ROA and EPS are the most widely used indicators to measure firm performance, mainly to reflect the efficiency of a company’s assets in generating income for the company’s shareholders.

Business model innovation is calculated by weighting six different indexes using principal component analysis and entropy weight method [102, 103]. It is a composite of the firm’s six variables (R&D expense ratio, Fixed and Intangible Assets Ratio, Customer Concentration, Main Income Revenue share, Total Asset Turnover, Efficiency of Labor), which is according to the structure of value creation, value proposition, and value capture innovation [104]. So, business model innovation can increase firm performance and should positive impact.

In this paper, the independent variable is mainly capital structure (CS), including total debt rate (TDTA), short-term debt rate (STDTA), and ownership concentration (OC10). The total debt rate, which indicates the level of debt, is obtained by comparing total debt to total assets [20, 40]. The level of a company’s total debt reflects its financing ability and the current level of risk it is taking, and it will have different impacts on the company’s operating decisions. We believe there is an optimal capital structure as the result of an equilibrium where the benefits of control are equal to the costs of bankruptcy [20, 53, 61], so the relationship between debt rate and firm performance or business model innovation should be U shape or inverted U shape.

The short-term debt rate represents the short-term debt to total asset ratio [20, 40, 41, 54, 105]. The level of a company’s short-term debt reflects the proportion of debt that needs to be repaid within one year. It is an essential reflection of the company’s risk exposure and promotes the flexibility and richness of operations. So, we propose that short-term debt will promote the firm’s performance and the business model innovation.

Ownership concentration (OC10), which is measured by the sum of the share rate of the top ten shareholders [20, 47, 48], reflects the dispersion of the shareholding ratios in one company and embodies the control of the major shareholders over the company and the mutual checks and balances between them. So, we expect a positive sign.

*C*_*it*_ are control variables that comprise:

Firm size (SIZE) is calculated as the natural logarithm of total assets at the end of each year, and firm size is correlated with capital structure and firm performance [4, 8, 68, 92, 105].

Firm age (AGE) indicates the operational maturity level of one firm. The age of a company is an indicator of the time it has been in operation. Scholars reduce the dispersion of company age by taking the logarithm of the difference between the observation year (2023) and the establishment year (the year the company was founded). This helps to reduce the variability of company age, making the data more reliable [10, 22, 23].

Non-debt tax shield (DEP) is the depreciation of a company’s fixed assets, and it is tax-deductible, like interest on debt. This type of factor is not only debt but also tax-deductible. It is measured by the depreciation of fixed assets divided by total assets [4, 105].

Board size of directors (BSIZE) generally uses the log of the number of directors on the board to measure [106, 107]. The size of the board of directors is often related to the level of corporate governance and impacts the company’s operating decisions and efficiency. Therefore, it is included as a control variable in this paper.

Independent directors ratio (INDDIR) is the ratio of the number of independent directors to the total number of directors on the board [4]. According to the Chinese Company Law, the number of independent directors on the board of a listed company shall not be less than one-third. Independent directors can also provide independent opinions to the board of directors to help the board of directors make correct decisions. The proportion of independent directors usually reflects a firm’s corporate governance level.

Quick ratio (QR) represents the firm’s solvency and is defined as current assets minus inventories divided by current liabilities [68, 105]. A higher quick ratio indicates that a company has a more vital short-term debt repayment ability. Generally, a quick ratio of 1.5 or more is considered reasonable. A quick ratio excessively low usually indicates that a company has a weak short-term debt repayment ability and may be in a liquidity crisis, which can affect corporate decision-making and operating performance.

All the variables are listed in Table 4.

**Table 4:** Definitions of the variables.

Indicators	Meaning	Variable measurement	Source
TDTA	Total Debt Asset Ratio	Total debt / Total assets	[108]
TDTA2	Square of TDTA	TDTA*TDTA	[108]
STDTA	Short-term Debt to Assets	Short-term / by total assets	[108]
OC10	Stock ownership concentration	Sum of share held rate of top ten shareholders	[20]
ROA	Return on Assets	Annual earnings / total asset value (main measure)	[108]
EPS	Earnings per Share	Net profit / Share	[20]
BMI	Business Model Innovation	Constructed using principal component analysis	[104]
BMIe	Business Model Innovation	Constructed using the entropy weight method	[104]
RD	R&D Expenses	R&D expenses / Operating income	[102]
FINTAN	Fixed and Intangible Assets	(Fixed + Intangible assets) / Total assets	[103]
CC	Customer Concentration	Percentage of sales revenue from top five customers	[103]
MIR	Main Income Revenue	Main Revenue / Operating income	[102]
TAT	Total Assets Turnover	Operating income / Total assets	[103]
EOL	Efficiency of Labour	Operating income / Total number of workers	[103]
SIZE	Firm Size	Log of ending total assets	[108]
AGE	Firm Age	Difference between 2023 and establishment year	[108]
DEP	Non-debt Tax Shield	Depreciation of fixed assets / Total assets	[4]
BSIZE	Board of Directors Size	Log of the number of directors on the board	[109]
INDDIR	Independent Directors Rate	Independent directors number / Directors number	[4]
QR	Quick Ratio	Current assets minus Inventories / Current liabilities	[109]

### 3.2 Estimation technique

This paper generally adopts a dynamic panel design and applies the generalized method of moments (GMM) to estimate the model. If the model is formulated using a dynamic approach, GMM is a proper estimation technique [110]. GMM helps to estimate a model when there is suspicion of unobservable data [111].

[112] used all possible lag variables as instrumental variables (there may be more instrumental variables than endogenous variables) for estimation, a method also known as differential GMM. In order to overcome problems such as the inability of differential GMM to estimate variables that do not change with time and the strong persistence of sequences. [113] returned to the horizontal equation before the difference and proposed the horizontal GMM estimation. [114] combined differential GMM and horizontal GMM for GMM estimation and proposed system GMM. The advantage of system GMM over differential GMM is that it can improve the efficiency of estimation and can estimate variables that do not change with time [114]. Sys-GMM estimation also corrects the simultaneous bias between the variable of interest and control variables [110, 111].

Proper instrument variables could help us to obtain unbiased results. Without valid instruments, reverse causality undoubtedly leads to biased estimates and results [112, 114]. Since sys-GMM uses more moment conditions, uses more information, and is more efficient in estimation, this paper will prioritize the use of system GMM when selecting estimation strategies.

The validity of the instruments is essential in the GMM estimator. So, two different but necessary tests will be used to guarantee they do not have the issues. First, difference-GMM and sys-GMM are valid on the premise that the error term ε does not have a serial correlation. Otherwise, it would lead to endogeneity problems. Therefore, it is necessary to test the second-order serial correlation of error terms [112]. The second test is over identifying test. Test whether the instrument variable is related to the error term and whether the instrument variable is exogenous. So, the Sargan test will be used after GMM regression [112–114].

### 3.3 Sample and data

We use the Chinese GEM high-tech listed companies’ data from the Wind database. The sample period is 2016–2022. There are 374 enterprises belonging to high-tech industries. The total number of listed companies in Chinese GEM is 1273(19/06/2023). Firstly, we delete enterprises that are not high-tech and were established after 2015. Secondly, the elements with serious missing data in the observation period (2016–2022) are removed. Thirdly, exclude the enterprises with ST. Fourthly, a few enterprises with missing values were supplemented manually by referring to the data in the annual reports. The number of enterprises involved in the model estimation is 374, and their data is applied for seven years from 2016 to 2022, and the total number of observations is 2583. Finally, to remove the influence of outliers on the model estimation results, the original data are shrunken at the 1% and 99% quantile of the above data.

We use six variables (RD/FINTAN/CC/MIR/TAT/EOL) to represent business model innovation. This indicator follows the structure and gives us a good framework and reference to measure business model innovation [104]. This thesis adopts the research framework and divides business model innovation into three dimensions: value creation innovation (RD and FINTAN), value proposition innovation (CC and MIR), and value capture innovation (TAT and EOL). Then, we use the principal component analysis method and entropy weight method to reduce the above six indicators into one indicator. BMI and BMIe are the indicators used to measure the business model innovation level of different firms.

First, principal component analysis is used to extract the common factors, the selected factor extraction criterion is eigenvalue ≥ 1, and the orthogonal varimax method is chosen to rotate the factors to obtain the rotated factor loadings matrix. Then, the composite evaluation value of business model innovation is obtained based on the factor score coefficient matrix and factor analysis table. The steps of the entropy weight method mainly include (1) standardization of all indicators, (2) calculation of the weight of indicator *j* in year *i*, (3) calculation of information entropy and redundancy of indicators, (4) calculation of indicator weight, and (5) calculation of comprehensive indicators.

Table 5 reports the descriptive statistics results of the variables used in this study. We find that the means and median of ROA are positive, although there is a minimum value of -31.26, the profitability of most sample enterprises is still strong, and the overall state is profitable. The mean of TDTA is 36%, which means that in China, for the GEM high-tech listed enterprises from 2016 to 2022, the average debt ratio is 36%, which is relatively modest and similar to the median. The minimum values of TDTA and STDTA are both 0.044, indicating that for some enterprises, there is only short-term debt and no long-term debt. The average value of OC10 is 51.5%, which is similar to median. However, the difference in ownership structure from 22.2% to 77% shows that the difference in ownership control in China’s capital market is still huge.

**Table 5:** Descriptive statistics.

Variable	N	Mean	p50	SD	Min	Max
ROA	2583	3.983	5.138	8.766	-31.26	23.43
BMI	2583	0.000	0.00600	0.513	-2.170	2.338
TDTA	2583	0.360	0.341	0.184	0.0440	1.049
STDTA	2583	0.297	0.279	0.158	0.0440	0.741
OC10	2583	0.515	0.520	0.128	0.222	0.770
SIZE	2583	21.80	21.74	0.847	20.11	24.30
AGE	2583	23.49	23	3.992	16	36
DEP	2583	8.343	6.384	6.975	0.519	37.06
BSIZE	2583	2.060	2.079	0.184	1.609	2.485
INDDIR	2583	38.30	37.50	5.482	33.33	57.14
QR	2583	2.383	1.564	2.366	0.435	14.55
EPS	2583	0.209	0.212	0.555	-1.973	1.995
BMIe	2583	0.283	0.277	0.0630	0.111	0.549

Table 6 presents the correlation matrix between the different variables. Most of the correlation values are not high enough. However, the correlation of TDTA and STDTA is 0.93, which means it is likely that the debts of plenty firms consist mainly of short-term debt, with little or no long-term debts. The correlation between QR and TDTA is -0.67, and with STDTA is -0.65. The correlation between ROA and EPS is 87%. However, this value does not matter because they are not simultaneously implemented in the same model.

**Table 6:** Correlation matrix.

	ROA	BMI	TDTA	STDTA	OC10	SIZE	AGE
ROA	1						
BMI	0.13***	1					
TDTA	-0.27***	0.23***	1				
STDTA	-0.26***	0.21***	0.93***	1			
OC10	0.25***	0.10***	-0.12***	-0.11******	1		
SIZE	0.08***	0.12***	0.41***	0.32***	-0.09***	1	
AGE	-0.0200	-0.0200	-0.0300	-0.0200	-0.0100	-0.0200	1
DEP	-0.0300	0.32***	-0.0200	-0.05**	0.0200	-0.19***	0
BSIZE	0.04*	0.04**	0.04**	0.06***	0.06***	0.08***	0.0200
INDDIR	-0.0300	-0.06***	0	-0.04*	-0.0200	-0.0300	-0.0200
QR	0.19***	-0.18***	-0.67***	-0.65***	0.08***	-0.31***	0.04*
EPS	0.86***	0.12***	-0.20***	-0.19***	0.22***	0.18***	-0.0300
BMIe	-0.0100	0.60***	0.08***	0.06***	-0.08***	-0.0100	-0.07***
	DEP	BSIZE	INDDIR	QR	EPS	BMIe	
DEP	1						
BSIZE	0.0300	1					
INDDIR	0.0100	-0.66***	1				
QR	-0.07***	-0.06***	-0.0100	1			
EPS	-0.07***	0.04**	-0.0200	0.13***	1		
BMIe	0.30***	-0.0100	0.06***	-0.04*	-0.04*	1	

Notes

* p < 0.1

** p < 0.05

*** p < 0.01

In order to avoid serious multicollinearity problems, a collinearity test is carried out. Variance Inflation Factor, or VIF, is the most widely used indicator to test the multicollinearity issue. If the value of VIF is more than 10 for the independent and control variables, a multicollinearity problem tends to exist in the model [110, 115]. Table 7 shows the results, in which the highest value of VIF is 8.57 and still less than 10. Therefore, we believe that although some variables have a high degree of correlation, the multicollinearity issue is not severe and could not be considered.

**Table 7:** Multicollinearity test.

Variable	VIF	Tolerance	Variable	VIF	Tolerance
TDTA	8.57	0.116618	BMIe	1.72	0.581297
STDTA	7.8	0.128234	SIZE	1.45	0.68872
BMI	1.92	0.521724	DEP	1.25	0.796945
QR	1.91	0.524644	EPS	1.24	0.805114
INDDIR	1.86	0.537889	OC10	1.12	0.890679
BSIZE	1.84	0.543861	AGE	1.01	0.989538
Mean VIF	2.76				

## 4. Results

This section reports and discusses the sys-GMM regression results using the basic model and the robustness analyses.

### 4.1 Results between capital structure and firm performance

Table 8 reports the estimated results from using return on assets (ROA) as a dependent variable and capital structure (CS) as independent variables, including total debt rate (TDTA), short-term debt rate (STDTA), and ownership concentration (OC10). Because we believe a complex relationship exists between debt ratio and firm performance, we include the square term of TDTA and the first-order lag item in the equation.

**Table 8:** Basic and the robustness test of capital structure and firm performance.

	(1)	(2)	(3)	(4)	(5)	(6)
	LOLS	LFE	LFE_DK	ROA	EPS	ROA_ro
L.ROA	0.341***	-0.153***	-0.153	0.242***		0.307***
	(11.21)	(-3.66)	(-1.10)	(2.70)		(5.20)
L2.ROA	0.116***	-0.280***	-0.280**	0.100		0.0872
	(3.87)	(-9.73)	(-2.84)	(1.50)		(1.59)
TDTA2	-44.91***	-40.05***	-40.05***	-73.46	-4.655	-89.97**
	(-4.19)	(-3.34)	(-7.49)	(-1.06)	(-1.43)	(-2.23)
L.TDTA2	39.70***	21.28	21.28**	47.28**	3.569**	42.22**
	(3.18)	(1.63)	(3.34)	(2.24)	(2.13)	(2.09)
TDTA	5.577	-10.59	-10.59	9.384	2.417	54.21
	(0.57)	(-0.89)	(-1.39)	(0.50)	(0.78)	(1.36)
L.TDTA	-0.129	-5.771	-5.771	25.46	-1.193	3.653
	(-0.01)	(-0.53)	(-0.57)	(0.38)	(-1.02)	(0.21)
L2.TDTA	-8.634*	-1.648	-1.648	-12.83	-0.713	-11.77*
	(-1.86)	(-0.28)	(-0.31)	(-0.72)	(-1.50)	(-1.78)
STDTA	-5.364	9.730	9.730	-12.89	0.933	15.40
	(-1.00)	(1.60)	(1.74)	(-0.67)	(0.87)	(1.01)
L.STDTA	15.33**	18.62***	18.62**	23.31	1.030**	14.77**
	(2.55)	(3.56)	(4.31)	(0.99)	(2.49)	(2.16)
L2.STDTA	-6.893	-2.623	-2.623	16.15*	-0.120	-3.919
	(-1.50)	(-0.47)	(-1.13)	(1.87)	(-0.24)	(-0.56)
OC10	8.850*	-1.177	-1.177	-0.767	0.0336	1.236
	(1.78)	(-0.23)	(-0.18)	(-0.08)	(0.06)	(0.16)
L.OC10	1.548	3.962	3.962	-19.09	0.359	10.99
	(0.23)	(0.72)	(0.54)	(-1.33)	(0.62)	(1.28)
L2.OC10	-2.811	-3.299	-3.299	2.308	0.0366	1.313
	(-0.74)	(-0.65)	(-1.17)	(0.24)	(0.09)	(0.18)
L.EPS					0.402***	
					(7.22)	
L2.EPS					0.124**	
					(2.38)	
_cons	-37.15***	-131.5***	0	-145.4	-5.131	-6.747
	(-4.78)	(-3.81)	(.)	(-0.89)	(-1.15)	(-0.11)
Entityfixed	Yes	Yes	Yes	Yes	Yes	Yes
YearFixed	Yes	Yes	Yes	Yes	Yes	Yes
Controls	Yes	Yes	Yes	Yes	Yes	Yes
Observations	1818	1818	1818	1818	1818	1611
AR (2) test				0.424	0.364	0.887
Sargan test				0.733	0.716	0.181

Notes

* p < 0.1

** p < 0.05

*** p < 0.01

z statistics in parentheses, Entityfixed: Entity Fixed; YearFixed: Year Fixed; Controls: Controls

In Table 8, column (1) shows the results of mixed OLS regression based on panel clustering without considering the dynamic panel properties, and column (2) shows the results of regression based on panel clustering and fixed effect model. Considering the potential heteroscedasticity or correlation issues, in column (3), the fixed effect model with Driscoll and Kraay robust standard error is adopted for estimation because Driscoll-Kraay standard errors are robust to very general forms of cross-sectional ("spatial") and temporal dependence when the time dimension becomes large [116]. The results of these three columns are all used for comparison. Column (4) is the basic model using the sys-GMM estimator and column (5) is a robustness analysis with EPS as the dependent variable. In column (6), we still use ROA as a dependent variable but delete the samples if the industry has less than five firms. We all use the cluster robustness standard error in columns (4) to (6).

The column (1) does not consider the individual fixed effect and may lose the individual characteristics. The estimated coefficients are consistent since columns (2) and (3) only use different standard errors. However, as mentioned, because subtracting the mean value from each variable results in a correlation between the explanatory variables and the error term, there are several cases where the coefficient sign of the variable is opposite to the theoretical estimate, so the fixed-effect model is inconsistent. There is "dynamic panel bias," and other estimation methods need to be introduced.

In column (4), we use the sys-GMM estimate and show positive and statistically significant memory effects from last year’s firm performance to the current year, and the coefficient is 0.242. The total debt ratio of the firm in the current year does not significantly impact the current year’s operating performance. However, the debt ratio of the previous year will have an impact on the operating performance of the current year, which also shows that the mechanism of the debt rate affecting the operating results of the enterprise has a certain "time lag." From the point of view of this paper, it is also logical because all the data in this paper are taken from the same time. It is reasonable that the state at the end of last year affects the result of this year. The regression results show that the total debt ratio at the end of the previous year will have a complex impact on firm performance, showing a U-shaped relationship; that is, with the increase of the debt ratio in the previous year, the business performance of the enterprise will decline, and when it reaches a certain level, the further increase of the debt ratio will significantly increase the firm performance [75, 76]. The coefficient of short-term debt is positive but has an insignificant effect on ROA. First-order lag of short-term debt rate has positive and statistically significant effects on ROA. This result is confirmed by the fact that the high level of last year’s short-term debt rate contributes more to this year’s firm performance. This conclusion also reflects that the impact of short-term debt on firm performance has a time lag, and short-term debt can promote firm performance improvement [59, 86]. Ownership concentration and its lag items have no significant effect on firm performance.

Column (5) is the result using EPS as the dependent variable for the robustness test. The square term of the total debt ratio is still not significant. At the same time, its first-order lag remains significantly positive, and the lag of the short-term debt ratio of the first order also has a significant positive impact on firm performance at the significant level of 5%. The degree of ownership concentration and its lagging terms are still not significant. In column (6), we narrow the samples to more typical industries.

Moreover, the result is not significantly different from the previous two columns. The coefficient of the ROA first-order lag increases from 0.249 in column (4) to 0.307. The coefficient of TDTA2 is negative and significant with firm performance. Furthermore, the first-order lag of TDTA2 and STDTA is still positive and significant with firm performance, which is consistent with columns (4) and (5). In columns (4) to (6), the AR test and Sargan test are all passed. So, from the results in Table 8, we can see that last year of total debt rate has a U-shaped correlation with this year’s firm performance, and last year’s short-term debt rate increases the firm performance of the current year.

### 4.2 Results between capital structure and business model innovation

Table 9 reports the estimated results between business model innovation and capital structure. We use BMI as the dependent variable of business model innovation and use BMIe in the robustness test. The dependent variable is capital structure (CS), still including the square of total debt rate (TDTA2), total debt rate (TDTA), short-term debt rate (STDTA), and ownership concentration (OC10). In columns (1) to (3), we still use ID clustering OLS regression, clustering robust standard fixed effect, and DK standard error fixed effect, and these columns are used for comparison. In columns (4) and (5), we use BMI and BMIe as dependent variables and use sys-GMM to test the correlation between capital structure and business model innovation. In the last two columns, we still adopt the method of limiting industries, excluding industries with less than five enterprises, and then use BMI and BMIe, respectively, for sys-GMM.

**Table 9:** Basic and the robustness test of capital structure and business model innovation.

	(1)	(2)	(3)	(4)	(5)	(6)	(7)
	LOLS	LFE	LFE_DK	BMI	BMIe	BMI_ro	BMIe_ro
L.BMI	0.544***	0.048	0.048	0.252**		0.258**	
	(17.82)	(1.19)	(0.55)	(2.39)		(2.04)	
L2.BMI	0.237***	-0.142***	-0.142*	0.0541		0.0470	
	(7.93)	(-3.89)	(-2.24)	(0.84)		(0.71)	
TDTA2	-0.702***	-0.725***	-0.725**	-1.875	0.092	-1.656	0.003
	(-3.08)	(-3.02)	(-4.23)	(-0.59)	(0.47)	(-0.74)	(0.01)
L.TDTA2	0.821***	0.471*	0.471**	1.439**	0.156**	1.498**	0.160**
	(2.89)	(1.96)	(3.73)	(2.35)	(2.43)	(2.01)	(2.06)
STDTA	0.514***	0.548**	0.548**	-0.0269	-0.095	0.090	-0.038
	(2.63)	(2.29)	(3.63)	(-0.02)	(-0.97)	(0.08)	(-0.30)
L.STDTA	-0.163	0.020	0.020	0.133	0.004	0.200	-0.001
	(-0.74)	(0.10)	(0.46)	(0.37)	(0.13)	(0.53)	(-0.02)
L2.STDTA	-0.370***	-0.150	-0.150	-0.156	0.013	-0.501	-0.025
	(-3.36)	(-1.23)	(-1.24)	(-0.46)	(0.40)	(-1.45)	(-0.75)
OC10	-0.152	-0.165	-0.165	-0.308	-0.049	-0.475	-0.084*
	(-0.72)	(-0.77)	(-1.90)	(-0.58)	(-1.06)	(-0.90)	(-1.75)
L.OC10	0.186	0.090	0.0896	0.269	0.019	0.479	0.054
	(0.67)	(0.41)	(0.78)	(0.71)	(0.62)	(1.35)	(1.42)
L2.OC10	0.101	-0.088	-0.088	-0.088	-0.025	0.139	-0.036
	(0.58)	(-0.44)	(-1.60)	(-0.26)	(-0.72)	(0.39)	(-1.03)
TDTA				1.921	-0.035	1.461	0.020
				(0.71)	(-0.21)	(0.76)	(0.10)
L.TDTA				-0.811	-0.137**	-0.795	-0.138**
				(-1.21)	(-2.21)	(-0.97)	(-1.97)
L2.TDTA				0.108	-0.005	0.350	0.030
				(0.36)	(-0.16)	(0.94)	(0.93)
L.BMIe					0.509***		0.488***
					(6.56)		(5.09)
L2.BMIe					0.022		0.029
					(0.56)		(0.68)
_cons	-0.061	-0.045	0	11.79	0.830	5.399	0.478
	(-0.24)	(-0.04)	(.)	(0.75)	(1.07)	(0.42)	(0.64)
Entityfixed	No	Yes	Yes	Yes	Yes	Yes	Yes
YearFixed	Yes	Yes	Yes	Yes	Yes	Yes	Yes
Controls	Yes	Yes	Yes	Yes	Yes	Yes	Yes
Observations	1818	1818	1818	1818	1818	1611	1611
AR (2) test				0.230	0.637	0.725	0.785
Sargan test				0.689	0.992	0.806	0.965

Notes

* p < 0.1

** p < 0.05

*** p < 0.01

z statistics in parentheses, Entityfixed: Entity Fixed; YearFixed: Year Fixed; Controls: Controls

The coefficient estimates in the first three columns are unstable, and even some coefficient signs are pretty opposite. In columns (4) to (7), the coefficient of first-order lag BMI or BMIe are positive and significant, and in columns (4) and (6), the coefficient values are 0.252 and 0.258, which have little difference, and in column (5) and (7), the coefficient values are both around 0.5 (0.509 and 0.488). Therefore, we believe enterprise business model innovation has some "inertia." That is, the degree of enterprise business model innovation in the previous year will significantly and positively affect the level of business model innovation in the current year, thus forming a "business model innovation chain."

The first-order lag TDTA2 is positive and significantly affects business model innovation in all seven columns, regardless of the variable estimate strategies used. In columns (4) and (6), although samples of different sizes are used, there is little difference between the two coefficients of regression results (1.439 and 1.498). In columns (5) and (7), the coefficients are 0.156 and 0.16, which also have little discrepancy. Moreover, the AR test and Sargan test are both passed. Therefore, we believe that the total debt ratio of the enterprise in the past period also presents a stable U-shaped relationship on the business model innovation in the current period because the change of capital structure also needs some time to affect the enterprise decision and business model innovation. However, the regression results show that this impact relation is stable and sustained. Short-term debt rate and ownership concentration do not influence business model innovation.

### 4.3 Results between business model innovation and firm performance

Table 10 demonstrates the estimated results between business model innovation and firm performance. We use BMI and BMIe as the independent variables and ROA and EPS as the dependent variables. Column (1) is the basic model using the sys-GMM estimator and columns (2) to (6) are robustness analyses. In column (2), we use EPS instead of ROA; in column (3), we replace BMI with BMIe. In columns (4) to (6), we use the same method or variables except to reduce the samples following the screening principles mentioned earlier.

**Table 10:** Basic and the robustness test of business model innovation and firm performance.

	(1)	(2)	(3)	(4)	(5)	(6)
DV	ROA	EPS	ROA	ROA	EPS	ROA
IV	BMI	BMI	BMIe	BMI_ro	BMI_ro	BMIe_ro
L.ROA	0.193**		0.104	0.212**		0.213***
	(2.45)		(1.19)	(2.22)		(2.92)
L2.ROA	0.0826		0.0520	0.115*		0.0934
	(1.59)		(0.87)	(1.67)		(1.56)
BMI	5.797***	0.293***		5.747***	0.359***	
	(4.26)	(3.77)		(3.84)	(4.05)	
L.BMI	0.0788	0.0542		0.517	0.0678	
	(0.07)	(0.97)		(0.40)	(1.07)	
L2.BMI	0.527	0.0514		-0.431	0.0345	
	(0.32)	(0.73)		(-0.22)	(0.37)	
L.EPS		0.276***			0.237***	
		(3.74)			(3.29)	
L2.EPS		0.0631			0.0761	
		(1.28)			(1.16)	
BMIe			23.37*			19.79*
			(1.70)			(1.84)
L.BMIe			11.75			32.13***
			(1.20)			(2.78)
L2.BMIe						26.45***
						(2.82)
_cons	-217.4	-8.416	-221.4	-257.6*	-6.895	-317.0*
	(-1.32)	(-1.20)	(-1.45)	(-1.67)	(-1.20)	(-1.95)
Entityfixed	Yes	Yes	Yes	Yes	Yes	Yes
YearFixed	Yes	Yes	Yes	Yes	Yes	Yes
Controls	Yes	Yes	Yes	Yes	Yes	Yes
Observations	1818	1818	1818	1611	1611	1611
AR (2) test	0.929	0.981	0.711	0.584	0.666	0.482
Sargan test	0.925	0.624	0.365	0.598	0.214	0.215

Notes

* p < 0.1

** p < 0.05

*** p < 0.01

z statistics in parentheses, Entityfixed: Entity Fixed; YearFixed: Year Fixed; Controls: Controls

As we can see, the first-order lag of firm performance is positive and significant, with the dependent variable in most columns. So, these results once again prove that past firm performance positively affects the aforementioned firm’s performance. Focus on the core variable and correlation of these models. The business model innovation level can significantly improve firm performance in all models. In most models, the first-order lag BMI or BMIe is insignificant with firm performance. In columns (1) and (4), no matter what the sample size, the coefficients are around 5.7 (5.797 and 5.747), which shows strong robustness. When we use EPS as the proxy variable of firm performance, just like columns (2) and (4) show, the coefficients are 0.293 and 0.359. Although these two values are not as robust as the results obtained by using ROA, they are also around 3.2. In columns (3) and (5), when we use BMIe as the independent variables, the coefficients are 23.37 and 19.79 (both around 20). Furthermore, the AR test and Sargan test are both passed. Based on our findings, we can conclude with a high degree of confidence that business model innovation has a significant positive impact on firm performance. This conclusion is consistent with our original hypothesis and the findings of previous studies [22–24, 32, 34, 96].

### 4.4 Mediation effect of business model innovation

According to the resource-based view (RBV) theory, different capital structures lead to different resource bases, leading to different decision-making, strategy changes, and new knowledge. These changes can be reflected in the innovation of business model elements [117, 118]. Therefore, any change in business model elements (such as technological innovation, product innovation, and team management innovation) can lead to business model innovation [32, 34, 39], ultimately improving firm performance.

It was found that business model innovation mediates in improving firm performance. We have already confirmed that the first-order lag of capital structure influences the current business model innovation level and that the current business model innovation level positively impacts firm performance. Therefore, business model innovation may mediate the relationship between capital structure and firm performance. To test this hypothesis, we conducted a Bootstrap Sobel test analysis with higher statistical efficacy [119–123].

We still use ROA and EPS as proxies for firm performance and BMI and BMIe as variables for business model innovation. As shown in Table 8, ownership concentration does not affect firm performance. Therefore, we only test whether business model innovation mediates the relationship between total debt to total assets (TDTA) and firm performance between short-term debt (STDTA) and firm performance. Table 11 shows the results of the Sobel test using the 1000 times bootstrap sampling. When testing the mediating effect of business model innovation on TDTA and firm performance, whether ROA or EPS represents firm performance, and whether BMI or BMIe represents business model innovation, the 95% confidence interval does not include 0. When testing the mediating effect of business model innovation on STDTA and firm performance, the 95% confidence interval includes 0. Therefore, it can be concluded that business model innovation mediates the relationship between total debt to total assets and firm performance but not between short-term debt and firm performance. This is also reflected in Table 9, because STDTA or its first-order lag is insignificantly correlated with business model innovation.

**Table 11:** Mediate effect test results.

IV	DV	MV	[95% conf. interval]		0 included
TDTA2	ROA	BMI	-46.827	-22.656	No
TDTA2	EPS	BMI	-3.220	-1.808	No
TDTA2	ROA	BMIe	-49.404	-25.119	No
TDTA2	EPS	BMIe	-3.333	-1.919	No
STDTA	ROA	BMI	-3.787	7.524	Yes
STDTA	EPS	BMI	-0.088	0.679	Yes
STDTA	ROA	BMIe	-2.239	8.627	Yes
STDTA	EPS	BMIe	-0.037	0.730	Yes

## 5. Discussion

This study investigates the impact of corporate capital structure and business model innovation on firm performance, utilizing a system-GMM approach with data from Chinese listed enterprises. It was found that the previous year’s performance positively influences the current year’s performance levels. This is attributed to the fact that better performance often signifies higher levels of innovation investment [124, 125], human capital [126], risk management practices [127], among others, all of which further contribute to enhancing firm performance.

This study reveals that the total debt ratio of a company at the end of the previous year has a complex impact on firm performance in current year, exhibiting a U-shaped relationship. The possible reason for this result is that when a firm has low debt, the tax shield effect of debt is minimal, and the costs associated with debt outweigh the tax benefits, hindering performance improvement. However, as the firm’s debt levels increase, the tax shield effect becomes more pronounced, thereby enhancing the firm’s value and net income. This aligns with the agency cost theory [51] and is supported by research findings from some scholars [75, 76]. With the increase of the short-term debt ratio, the firm performance also increases correspondingly. This is consistent with some research [18, 20, 59, 86, 128]. Short-term debt is the primary source of debt, just like [20] found that listed companies in China prefer short-term debt financing. The higher value of the short-term debt ratio usually leads more resources in one short period which will lead to better performance.

Furthermore, the research findings of this study did not reveal a significant relationship between equity structure and firm performance. Neither the current year nor previous years showed a significant impact on the performance of the current year. This could be attributed to the use of data from Chinese Growth Enterprise Market (GEM) companies, where the top ten shareholders in GEM companies exhibit high concentration and limited diversity in equity ownership. Additionally, the influence of equity structure is partially reflected in the size of the board of directors. As a result, the positive impact relationships found by scholars are not confirmed in this study [20, 47, 48].

The study reveals that business model innovation in the previous year significantly and positively influences the level of business model innovation in the current year and firm performance. [129, 130] argue that the only way to enhance organizational performance is through a bricolage of resources. This is because, according to the resource-based view theory and dynamic capabilities, the input and uniqueness of organizational resources determine the competitive advantage and performance of a firm. Business model innovation necessitates resource input, and these innovations and inputs often cannot be completed in a short timeframe but rather constitute a continuous dynamic process, giving rise to the "inertia" of business model innovation. Business model innovation improves the efficiency of decision-making in the value system, breaks down traditional barriers, changes the decision path, and achieves value addition in the marketplace than previous models [23]. Excellent business model innovation is a terrific way to improve competitive advantage and create benefits, which can lead to better firm performance [131, 132]. Therefore, for enterprises, unique and sustainable business model innovation is a crucial way to develop dynamic capabilities and competitive advantages. Once a company enters the "lane" of business model innovation, its advantages will lead to sustained improvements.

An important innovation in this study is the exploration of the relationship between capital structure and business model innovation, as no studies have been found investigating the impact of business model innovation from capital structure. The study finds that the total debt ratio of the previous period also exhibits a significant U-shaped relationship with business model innovation in the current period. This relationship is attributed to the increasing share of corporate debt in the capital structure of the past year, which gradually raises the risk of bankruptcy for the company. At this point, decision-makers within the company may lean towards adopting conservative operational and managerial measures, thereby suppressing the level of business model innovation. However, as the level of debt increases to some certain levels, the benefits of the debt tax shield become more pronounced, offsetting debt expenses and other costs [51, 76]. This provides companies with greater autonomy for innovation and potential, leading to higher levels of business model innovation. Moreover, as the amount of debt repayment increases, companies have a stronger drive and necessity for model innovation, further enhancing the level of business model innovation.

Furthermore, as discovered earlier in this study, business model innovation positively enhances firm performance, while capital structure has a U-shaped impact on business model innovation. Therefore, capital structure can influence firm performance by affecting business model innovation. Theoretically, according to the resource-based view theory, firms must acquire and control some resources and capabilities [133, 134] to achieve a competitive advantage. How much resources can be invested is primarily influenced by capital structure. Firms have responsibility to continuously develop the variety and adaptability of the resources to gain competitive advantage. Capital structure influences and provides different resources and bringing business model innovation undoubtedly. Business model innovation also require resource-based innovation to gain sustainable competitive advantage, which will lead to better firm performance.

## 6. Conclusion

This study aims to examine the impact of capital structure and business model innovation on firm performance in China. The main reason is that there are more and more high-tech enterprises in China, attracting the attention of capital markets. By clarifying the impact of different financing channels on their operating performance, firms can continuously adjust their capital structure and improve their operating performance. At the same time, with the increasing number of business model innovation cases in China, it is essential to clarify the transmission mechanism of business model innovation in the capital structure and firm performance and to supplement the theoretical research on this issue, which will provide Chinese high-tech enterprises with reference opinions. Therefore, this paper uses data from GEM-listed high-tech enterprises from 2016 to 2022 and adopts the sys-GMM method.

The study found that capital structure has a lag effect on enterprise performance and a noticeable "time lag effect." The total debt ratio in the last period significantly nonlinearly impacts this period’s firm performance and business model innovation level, presenting a U-shaped relationship. Enterprises’ first-order lag short-term debt ratio can effectively improve current firm performance. Ownership concentration has insignificant effect on firm performance and business model innovation. The higher the level of business model innovation in the current period, the better the firm performance. The extent to which a company innovated its business model in the previous year has a significant positive impact on the level of business model innovation in the current year. At the same time, this paper also verifies that business model innovation does exist in the mediating effect between enterprise capital structure and its performance.

The practical implications of this study lies in the following points: (1) Firms can adjust their financing structure based on the research findings in this study. Control the pace of debt financing, especially balance the relationship between financing risk and tax shield, and quickly escape the lowest point of the capital structure effect, and turn to the accelerating growth half of the curve, and use the positive effect of debt ratio on firm performance. (2) Firms can also take advantage of short-term debt to positively impact their performance and enhance their capacity for short-term debt financing. The government should provide enterprises with smoother, barrier-free short-term financing channels and basic guarantees to help them constantly improve their operating level. (3) Encourage firms to innovate their business models and take advantage of the direct effect of business model innovation on improving firm performance and the indirect effect of capital structure adjustment released through business model innovation, encourage and support enterprises to break through the existing business model, and improve enterprise innovation tolerance.

This paper also has some limitations, mainly involving a short sample period, all of the selected enterprises are Chinese listed companies, and the heterogeneity analysis of enterprises with different characteristics and the depth of research on business model innovation.

Future research can be further explored in the following fields: (1) Expand the sample size and extend the research period, and strive to include a variety of types of enterprises in China and other regions of the world to verify the universality of the above conclusions; (2) Discuss the classification and grouping of enterprises in different maturity, region, industry or scale to verify the robustness or difference of the relationship between enterprise capital structure, business model innovation and firm performance in different groups; (3) Conduct in-depth discussions on the mediating mechanism of business model innovation, and what kind of capital structure will make business model innovation more familiar and efficient. These are all worth further research and analysis.

## Supporting information

S1 File Research data. (XLSX)
